# Incidence and Associated Factors of HIV Drug Resistance in Chinese HIV-Infected Patients Receiving Antiretroviral Treatment

**DOI:** 10.1371/journal.pone.0062408

**Published:** 2013-04-30

**Authors:** Hui Xing, Xia Wang, Lingjie Liao, Yanling Ma, Bin Su, Jihua Fu, Jianmei He, Lin Chen, Xiaohong Pan, Yonghui Dong, Wei Liu, Jenny H. Hsi, Liting Yang, Yuhua Ruan, Yiming Shao

**Affiliations:** 1 State Key Laboratory for Infectious Disease Prevention and Control, and National Center for AIDS/STD Control and Prevention, Chinese Center for Disease Control and Prevention, Beijing, China; 2 Yunnan Center for Disease Control and Prevention, Kunming, China; 3 Anhui Center for Disease Control and Prevention, Hefei, China; 4 Shandong Center for Disease Control and Prevention, Jinan, China; 5 Hunan Center for Disease Control and Prevention, Changcha, China; 6 Shenzheng Center for Disease Control and Prevention, Shenzheng, China; 7 Zhejiang Center for Disease Control and Prevention, Hangzhou, China; 8 Xinjiang Center for Disease Control and Prevention, Urumqi, China; 9 Guangxi Center for Disease Control and Prevention, Nanning, China; The University of Hong Kong, China

## Abstract

**Background:**

A critical indicator of the future success of highly active antiretroviral therapy (HAART) is the incidence of HIV drug resistance, which has not been studied in China on the national scale.

**Methods:**

HIV drug resistance baseline survey was conducted in the eight provinces with the largest numbers of patients on HAART in 2009, and a prospective cohort study with 12-month follow-up was completed in 2010. Patients completed an interviewer-administrated questionnaire and provided blood for CD4+ T-lymphocyte count (CD4 count), HIV viral load (VL), and HIV drug resistance genotyping. Factors associated with incidence of HIVDR were identified by Cox regression analysis.

**Results:**

The overall prevalence of HIV RNA ≥1000 copies/ml and HIVDR at baseline was 12.4% and 5.6%, respectively. Incidence of HIVDR in the one year follow-up was 3.5 per 100 person years. Independently associated factors were started treatment with a didanosine-based regimen, received care at township hospital or village clinic, low baseline CD4 counts, and high baseline VL.

**Conclusions:**

The incidence of HIVDR in China was higher than that of some developed countries. China urgently needs to provide comprehensive education and training to doctors at village clinics and township hospitals to improve quality community-based care and treatment.

## Introduction

Since the introduction of combination drug regimens to treat human immunodeficiency virus (HIV) infection, known as highly active antiretroviral therapy (HAART), the rates of HIV-related morbidity and mortality have been markedly reduced. [1], [2] However, the presence of antiretroviral drug resistance mutations in the infecting viruses may hamper the effectiveness of antiretroviral treatment (ART) because the mutations reduce the chances of full viral suppression. The increasing use of ART would lead to an increase in the incidence and prevalence of drug resistance especially in developing countries under WHO guidelines [3]. Previous studies on the prevalence of HIV drug resistance (HIVDR) in China and their associated factors [4], [5] have served as assessments of the HIVDR consequences of China’s National Free Antiretroviral Treatment Program (NFATP). However, incidence of HIVDR is also a critical indicator of the future success of HAART but currently remains poorly studied in China. A few studies have investigated HIVDR incidence in limited risk populations, which showed that the crude incidence of both multidrug resistance and full-drug-class has decreased over time. [6]–[8] In this study, we aim to evaluate the incidence rate of HIVDR in China as well as to identify their associated factors.

## Methods

### Study Design and Study Participants

In 2009, the baseline survey on HIV drug resistance was conducted in the eight provinces in China with the largest numbers of patients on ART under the NFATP. The county in each province with the most patients was selected to receive the survey, and up to 250 patients were recruited for each province. If not enough eligible patients were found in the county, the county with the next largest number of patients was selected. All patients who received treatment from 2005 onwards were chronologically contacted for recruiting to the study; detailed subject recruitment procedure has been previously described. [4] The eligibility criteria include: receiving HIV antiretroviral therapy through NFATP from 2005 to 2009, being 18 years or older, and willingness and consent to participate. Treatments in the NFATP were first-line ART regimens consisting of 2 NRTIs [azidothymidine (AZT)+didanosine (DDI) or stavudine (D4T)+lamivudine (3TC)] and one NNRTI [nevirapine (NVP) or efavirenz (EFV)]. AZT, D4T, DDI, and NVP are generically produced in China, whereas 3TC and EFV are branded drugs which became available in 2005. All subjects provided written informed consent to participate in this study. The institutional review board (IRB) of the NCAIDS, China CDC approved this study.

Following the baseline survey, all patients were followed up one year later in 2010 to evaluate the incidence of HIV drug resistance (HIVDR). The survey sites were (outside parenthesis, provinces; inside parentheses, counties or municipalities): Yunnan (Long chuan), Anhui (Jieshou and Linquan), Shandong, Hunan (Hengyang), Guangdong (Shenzheng), Zhejiang (Hangzhou, Ningbo and Wenzhou), Xinjiang (Yining), and Guangxi (Hezhou). Shandong province has fewer patients who are dispersed throughout the province, hence patients were recruited from the whole province as opposed to in one county only.

### Data Collection

In the survey, an interviewer-administrated questionnaire interview was conducted to collect demographic data and data on ART treatment. Demographic variables include height, weight, ethnicity, education, residency, occupation, average monthly family income, and residency status (permanent vs. migrant). Treatment and behavior variables include initial treatment date, spouse ART status, taking traditional Chinese herbal medicines, receiving counseling and instructions on ART use (currently and before starting ART), clinical symptoms in the recent month, recent sexual behavior, recent alcohol use, recent drug, source of ART drug distribution, interval of refilling drug in the past month, treatment termination date, and reasons for terminating treatment. The variables on self-reported ART adherence include missed doses in the past month, and the proportion of medicines taken on time in the past month. Venous blood specimen samples were also collected for testing CD4^+^ T-lymphocyte count (CD4 count), HIV viral load, and HIVDR mutations.

### Laboratory Analysis

CD4 count was measured within 24 hours by flow cytometry in the local CDCs and was quality assured by the National HIV Reference Laboratory. Plasma was separated by centrifugation and stored at −80°C, then they were transported to NCAIDS through cold chain. HIV viral load and drug resistance mutation tests were performed at the National Center for AIDS/STD Control and Prevention (NCAIDS), China CDC. Plasma HIV-1 RNA copy was quantified with real-time Nucleic Acid Sequence Based Amplification (NASBA) (NucliSense Easy Q, BioMerieux, France) or COBAS (Roche Applied Science, Germany) according to the manufacturers’ protocols. In samples with viral load ≥1,000 copies/ml, HIV drug resistance genotyping was carried out by an in-house polymerase chain reaction (PCR) protocol as previously described. [9], [10] The HIV-1 pol gene (protease, amino acids 1–99; and part of reverse transcriptase, amino acids 1–252) was amplified. For analyzing HIV-1 drug resistance mutations, each sequence was compared with the subtype B consensus sequence in the Stanford HIV Drug Resistance Database (http://hivdb.stanford.edu) and was interpreted using the HIV db program. We included mutation results that conferred low-, intermediate-, and high- level resistance. [11].

### Statistical Analysis

Primary outcome variables: we defined a case of drug resistance as the combined condition of having a HIV viral load ≥1,000 copies/ml and displaying genotypic HIVDR mutation(s). We used Cox proportional hazard models to evaluate hazard ratios of HIVDR incidence. Time zero was defined as the enrollment date at the surveys, and incidence of HIVDR was defined as those who developed resistant mutations during the one year interval before follow-up. Variables that were significantly (*P*≤0.05) associated with death in the univariate analysis were considered for inclusion in multivariate Cox regression models. All tests of significance were two-sided, with a *P*-value ≤0.05.

## Results

### Demographic Characteristics

The baseline cross-sectional survey in 2009 included 2192 patients, among whom 2005 were followed up in 2010, 46 patients died, Of the remaining136 patients were not retained, 6 transferring out, 50 moving out of the area, and 80 losing to follow-up. The demographic and disease characteristics of the followed up patients are shown in Table 1. These include: 62.3% was male, mean age was 38.7±9.9, 68.3% was married, 38.8% had up to primary school education or less, 39.5% were farmers. The patients were primarily infected through sexual contact (55.1%), drug injection (23.0%) and blood/plasma transmission (15.6%).

**Table 1:** Baseline characteristics of HIV patients in the study.

Variable	Number	Percentage (%)
Total	2192	
Sex		
Male	1365	62.3
Female	827	37.7
Age in years: mean (SD), range	38.7 (9.9), 32–44	
Married		
Yes	1497	68.3
No	695	31.7
Education		
Illiterate	273	12.5
Primary school	577	26.3
Middle school	839	38.3
Junior high school or more	503	23.0
Occupation		
Farmer	1109	50.6
Other	1083	49.4
HIV transmission route		
Sexual contact	1208	55.1
Blood/plasma transmission	341	15.6
Drug injection	505	23.0
Other	138	6.3
Initial ART regimen		
AZT/D4T+DDI+NVP/EFV	114	5.2
AZT/D4T +3TC+NVP/EFV	2015	91.9
Other	63	2.9
Baseline ART regimen(2009)		
AZT/D4T+DDI+NVP/EFV	31	1.4
AZT/D4T +3TC+NVP/EFV	1986	90.6
Second-line regimens	133	6.1
Other	42	1.9
Duration of HAART treatment (months)		
0–12	813	37.1
13–24	556	25.4
25–36	393	17.9
>37	430	19.6
Baseline CD4(2009)		
<200	405	18.5
200–349	479	21.9
350–499	764	34.9
≥500	544	24.8
Baseline viral load ≥1000 copies/ml(2009)	272	12.4
HIV drug resistance(2009)		
Resistance to any drugs	123	5.6
Resistance to NNRTIs	115	5.3
Resistance to NRTIs	96	4.4
Resistance to NNRTIs and NRTIs	90	4.1
Resistance to PIs	6	0.3

Initial ART regimens (the regimen was used when the treatment was started) used were AZT/D4T+DDI+NVP (5.2%), AZT/D4T +3TC+NVP (72.2%), AZT/D4T +3TC+EFV (19.7%), and others (2.9%). However, in 2009, only 1.4% of patients still received DDI based regimens and 6.1% had been switched to second-line regimens. At the time of the baseline survey, the median duration of treatment was 17.6 months (interquartile range [IQR], 8.3–31.5). The mean CD4 count was 341.6 cells/µl, and the proportions of patients with CD4 count of 0–199, 200–349, and ≥350 cells/µl were 40.3%, 34.9% and 24.8%, respectively. The great majority of patients (1920/2192, 87.6%) had plasma HIV viral load <1,000 copies/ml. Among the patients with virologic failure, 123 (45.2%) had resistance mutations identified, including 90 (33.1%) with dual-class resistance.

Among the 2005 patients followed up in 2010, the mean CD4 count was 384.6 cells/µl, and the proportions of patients with CD4 count of 0–199, 200–349, ≥350 cells/µl were 23.2%, 31.4% and 45.4%, respectively. Approximately the same proportion of patients, 89.2% (1785/2002), had plasma HIV viral load <1,000 copies/ml. The incidence rate of death was 2.0 per 100 person years, with 46 patients having died during the follow-up period. Among patients retained at the 2010 follow-up and who had no HIVDR mutations in 2009, the incidence of resistance to any type of HIV drugs, as well as to NNRTIs, NRTIs, and PIs alone, were 3.5 per 100 person years (64/1837.3), 3.4 (63/1837.3), 2.6 (47/1837.3), 0.1 (1/1837.3), respectively, and 2.4% (46/1930) were resistant to both NRTIs and NNRTIs (Table 2). The most common NNRTI mutations were K103 and Y181 and the most common NRTI mutations were M184 and D67.

**Table 2:** HIVDR mutations among patients with drug resistance.

			Incidence	
Mutations			Number	%
Total			64	100.0
NRTIs			47	73.4
V75A/M/T			1	1.6
L74I/V			2	3.1
L100I			2	3.1
L210W			2	3.1
Q151L/M			2	3.1
M41L			3	4.7
T215C/D/F/I/S/Y			6	9.4
K70E/R			8	12.5
D67G/N			12	18.8
M184I/V			46	71.9
NNRTIs			63	98.4
M230L			1	1.6
A98G			2	3.1
P225H			3	4.7
Y188C/L/H			3	4.7
F227L			4	6.3
V106A/M			8	12.5
K101E/H/P			13	20.3
G190A/S			20	31.3
Y181C/V			22	34.4
K103H/N/S/T			25	39.1
PI			1	1.6
M46I			1	1.6
I54V			1	1.6
L76V			1	1.6
V82F			1	1.6

The risk factors for incidence of HIVDR were assessed through a Cox regression model (Table 3). The four factors that remained independently associated in the adjusted model were: initial ART regimen, ART drug distribution institute, baseline viral load, and baseline CD4 count. Those used AZT/D4T+DDI+NVP were 3.1 fold (95% CI 1.1–9.1) more likely to develop HIVDR compared to those used AZT/D4T +3TC+EFV, and those who received ART drugs in village clinics or township hospitals were 2.0 fold (95% CI 1.1–3.5) more likely to develop HIVDR than those who received treatment in county hospitals or CDCs. Patients with baseline viral load ≥1000 copies/ml were 5.9 fold (95% CI 3.2–10.6) more likely to develop HIVDR than those whose baseline viral load was less than1000 copies/ml. Compared to patients with baseline CD4 counts of ≥350/µl, patients with 0–199 cells/µl were 2.3 times (95% CI 1.2–4.5) more likely to develop HIVDR, and those with CD4 counts of 200–349 cells/µl were 1.6 times (95% CI 0.8–3.1) more likely to develop HIVDR.

**Table 3:** Factors associated with incidence of drug resistance in 2010.

Variable	Number	HIVDR	Person year	Incidence/100 personyear	HR (95% CI)	*P-value*	Adjusted HR(95% CI)	*P-value*
Total	1893	64	1837.3	3.5				
Sex								
Male	1166	47	1128.3	4.2				
Female	727	17	709.0	2.4	0.6(0.3,1.0)	0.07		
Age								
≤30	376	12	366.1	3.3				
31–40	862	33	842.2	3.9	1.3(0.6,2.4)	0.50		
41–50	422	15	406.0	3.7	1.2(0.5,2.5)	0.70		
>50	233	4	223.1	1.8	0.7(0.2,2.1)	0.49		
Married								
Yes	1309	43	1276.0	3.4				
No	584	21	561.4	3.7	1.1(0.7,1.9)	0.65		
Education								
Junior high school or more	1172	36	1140.1	3.2				
Primary school or less	721	28	697.3	4.0	1.2(0.7,1.9)	0.58		
Occupation								
Farmer	987	32	947.8	3.4				
Other	906	32	889.6	3.6	1.3(0.8,2.2)	0.27		
Monthly income per person within the family (RMB)								
<400	875	39	861.5	4.5				
≥400	1018	25	975.8	2.6	0.8(0.5,1.3)	0.29		
Spouse receives ART								
No	1452	53	1405.5	3.8				
Yes	441	11	431.8	2.5	0.7(0.4,1.3)	0.28		
HIV transmission route								
Sexual intercourse	1084	34	1056.7	3.2				
Drug injection	393	17	387.9	4.4	1.1(0.6,2.0)	0.73		
Blood donation or transfusion	301	10	281.2	3.6	1.3(0.7,2.7)	0.42		
Other	115	3	111.5	2.7	0.9(0.3,3.1)	0.91		
Initial ART regimen								
AZT/D4T +3TC+EFV	376	10	362.0	2.8				
AZT/D4T+DDI+NVP	85	6	79.2	7.6	4.1(1.5,11.3)	0.01	4.5(1.6,12.6)	<0.01
AZT/D4T +3TC+NVP	1375	48	1343.8	3.6	1.1(0.5,2.1)	0.9	0.8(0.4,1.7)	0.58
Other	57	0	52.4	0.0	–	–	–	–
Baseline ART regimen(2009)								
AZT/D4T +3TC+EFV	569	19	549.9	3.5				
AZT/D4T+DDI+NVP	33	2	32.3	6.2				
AZT/D4T +3TC+NVP	1187	39	1160.9	3.4				
Second-line regimen	49	1	43.6	2.3				
Other	55	3	50.7	5.9				
Duration of ART (year)								
0–12	694	27	660.8	4.1				
13–24	477	18	467.0	3.9	0.7(0.4,1.3)	0.29		
25–36	354	8	349.6	2.3	0.4(0.2,0.8)	0.01		
>37	368	11	360.0	3.1	0.5(0.2,1.0)	0.04		
Missed doses in the past month								
No	1838	59	1786.2	3.3				
Yes	92	5	85.7	5.8	2.3(0.9,5.9)	0.07		
Taking drugs on time								
<90%	73	5	70.9	7.1				
≥90%	1820	59	1766.4	3.3	0.5(0.2,1.2)	0.12		
ART drug distribution institute								
County hospital or CDC	1525	44	1483.6	3.0				
Village clinic or township hospital	368	20	353.7	5.7	1.6(0.9,2.7)	0.10	1.8(1.0,3.3)	<0.05
Interval of refilling drug in the past month								
Less than one month	1081	36	1026.1	3.5				
More than one month	812	28	811.2	3.5	0.8(0.5,1.3)	0.37		
CD4 cell counts at baseline(2009)								
≥350	797	18	785.5	2.3				
200?350	647	23	626.7	3.7	2.1(1.1,3.9)	0.02	2.0(1.1,3.7)	0.03
<200	449	23	425.1	5.4	4.1(2.1,7.8)	<0.01	3.5(1.8,6.9)	<0.01
Viral Load at baseline(2009)								
VL<1000	1773	45	1724.2	2.6				
VL≥1000	120	19	113.1	16.8	9.5(5.5,16.5)	<0.01	7.1(4.0,12.7)	<0.01

## Discussion

In this one-year prospective follow-up survey of HIVDR across eight provinces in China from January 2009 to December 2010, we found that the overall prevalence of HIVDR at baseline was 5.6%, virtually all with NNRTI mutations and three-fourths with NRTI mutations, which is comparable to proportions observed in other countries. [3], [12] The incidence of HIVDR during the one year follow-up was 3.5 per 100 person years, and NNRTI mutations and NRTI mutations were 3.4/100 person years and 2.6 per 100 person years, respectively. Factors independently associated with incidence of HIVDR were: initial treatment with a didanosine (DDI)-based regimen; receiving care at township hospital or village clinic; low baseline CD4 cell counts; and high baseline viral load.

An important concern for widespread ART use is the potential for emerging HIVDR mutations given improperly administered regimens and lack of drug adherence support in resource-limited settings. Our results here revealed that the HIVDR incidence rates in China are higher than those of a number of countries. An ecological study among ART treated patients in British Columbia, Canada reported that the incidence of HIVDR decreased dramatically from 1.73 per 100 person-months of therapy in 1997 to 0.13 per 100 person-months of therapy in 2008. [7] A study in Denmark showed that among 1829 treatment-naïve patients who initiated ART in or after 1998, the incidence of NRTIs and NNRTIs were 0.59 and 1.06 per 100 person-years. [6] In Portugal, a study showed that the annual incidence of HIVDR decreased from 5.7% in July 2001 to 2.7% in July 2006 in Portugal based on 3394 viral isolates. [8] It is therefore important for policymakers and care providers to address the factors driving China’s relatively high HIVDR incidence rates.

The first risk factor we identified is the use of DDI-based regimens, which resulted in higher HIVDR incidence compared with lamivudine (3TC)-based regimens. Previous studies have reported that DDI-based regimens are associated with higher rates of emergence of liver disease, [13] virologic failure, [14] prevalence of HIVDR [15]–[17] and mortality. [18] It is possible that because DDI is associated with more side effects compared with 3TC-based regimens, patients are less likely to stay adherent to drug intake and clinical visits. [18] The World Health Organization (WHO) suggested that DDI had serious constraints for use in first-line regimens because of toxicities and cost. [19] Although DDI is not recommended in the NFATP first-line cART regimens and few patients still use it, health care providers in China should pay close attention to the patients whose initial regimens contain DDI and has switched to other regimens.

Secondly, our findings show that patients who received care in rural village clinics or township hospitals were significantly more likely to experience incident HIVDR than those treated at county-level CDCs or hospitals staffed by trained physicians. Patients with low income are also more likely to develop drug resistance. As our previous study reported, patients in rural or lower-income regions in China have significantly lower levels education and socio-economic status, and health care providers have less advanced technical infrastructure and capacity. [20] It is likely that patients cannot properly adhere to complex treatment regimens without adequate assistance, and that improper use of these drugs by health care systems with low infrastructure will blunt their effectiveness and favor the emergence of antiretroviral resistance. [21] China urgently needs to improve health education among patients and training for doctors at village clinics and township hospitals to provide quality community-based care and treatment.

Finally, we found that lower baseline CD4 cell count and higher baseline viral load are significantly associated with incidence of drug resistance. Our results are consistent with previous studies reporting that initiating HAART at higher CD4 cell counts may decrease the risk of developing drug resistance [22], [23] and that higher baseline viral load was a major predictor of drug resistance. [24] HIV viral load and CD4 cell count are the primary clinical indicators that should be used to guide the initiation of antiretroviral therapy and subsequent changes in therapy. It is also consistent with other findings that starting antiretroviral therapy earlier yields better clinical outcomes for survival. [25], [26] At the start of China’s NFATP in 2002, treatment was provided for patients with CD4 count <200 cells/µl; in 2008, the eligibility criterion was changed to 350 cells/µl to improve the outcomes of treatment. Care must be taken to continuously monitor NFATP patients for emerging HIVDR in order to assess potential needs for amending these eligibility indicators.

In conclusion, Chinese policymakers and care providers need to consider the relatively high incidence of HIVDR in China and address the related factors, and provide comprehensive education and training to community health workers or nurses to improve health care quality.
